# Recent Transmission of Tuberculosis — United States, 2011–2014

**DOI:** 10.1371/journal.pone.0153728

**Published:** 2016-04-15

**Authors:** Courtney M. Yuen, J. Steve Kammerer, Kala Marks, Thomas R. Navin, Anne Marie France

**Affiliations:** Division of Tuberculosis Elimination, Centers for Disease Control and Prevention, Atlanta, Georgia, United States of America; University of Minnesota, UNITED STATES

## Abstract

Tuberculosis is an infectious disease that may result from recent transmission or from an infection acquired many years in the past; there is no diagnostic test to distinguish the two causes. Cases resulting from recent transmission are particularly concerning from a public health standpoint. To describe recent tuberculosis transmission in the United States, we used a field-validated plausible source-case method to estimate cases likely resulting from recent transmission during January 2011–September 2014. We classified cases as resulting from either limited or extensive recent transmission based on transmission cluster size. We used logistic regression to analyze patient characteristics associated with recent transmission. Of 26,586 genotyped cases, 14% were attributable to recent transmission, 39% of which were attributable to extensive recent transmission. The burden of cases attributed to recent transmission was geographically heterogeneous and poorly predicted by tuberculosis incidence. Extensive recent transmission was positively associated with American Indian/Alaska Native (adjusted prevalence ratio [aPR] = 3.6 (95% confidence interval [CI] 2.9–4.4), Native Hawaiian/Pacific Islander (aPR = 3.2, 95% CI 2.3–4.5), and black (aPR = 3.0, 95% CI 2.6–3.5) race, and homelessness (aPR = 2.3, 95% CI 2.0–2.5). Extensive recent transmission was negatively associated with foreign birth (aPR = 0.2, 95% CI 0.2–0.2). Tuberculosis control efforts should prioritize reducing transmission among higher-risk populations.

## Introduction

Cases of tuberculosis disease may occur as a result of recent transmission from an infectious case or via reactivation of remotely acquired latent infection. Cases resulting from recent transmission are particularly concerning from a public health standpoint because they represent the possibility of ongoing transmission from unrecognized infectious cases and the presence of recently infected contacts who would benefit from preventive therapy. Furthermore, when recent transmission of tuberculosis is undetected or unchecked, outbreaks can occur. Thus, identifying populations at risk for recent transmission, and specifically populations at higher risk for the type of extensive recent transmission associated with outbreaks, could help guide tuberculosis control efforts.

Genotype-based methods can be used to estimate recent transmission, as cases associated by recent transmission are more likely to share a genotype than cases caused by reactivation [1]. Previous analyses have described patient characteristics associated with genotypic clustering, using shared genotypes within a geographically defined population as an indicator for recent transmission [2, 3]. However, not all cases with a shared genotype are actually related by recent transmission [1], and most studies that use clustering as an indicator for recent transmission have not validated clustering results with field epidemiology to verify the likelihood of transmission. To address this concern, a method that uses genotype as well as temporal, spatial, clinical, and demographic factors to determine cases attributable to recent transmission was developed and validated by field epidemiology [4]. This “plausible source-case” method was the most accurate of multiple methods evaluated, and an accuracy of 94% was calculated given the range of prevalence of recent transmission expected in the United States.

To describe the epidemiology of tuberculosis cases resulting from recent transmission in the United States, we attributed cases to recent transmission by applying the plausible source-case method to routinely collected surveillance data. In addition, we sought to determine whether populations in which transmission is limited differ from populations in which uncontrolled tuberculosis leads to extensive transmission.

## Methods

### Case Inclusion and Classification

We used routinely collected data from the U.S. National Tuberculosis Surveillance System (NTSS) and the National Tuberculosis Genotyping Service (NTGS). Since 2004, NTGS has performed universal genotyping of culture-positive tuberculosis cases; genotyping has been based on spacer oligonucleotide typing (spoligotyping) and 24-locus mycobacterial interspersed repetitive unit variable number of tandem repeats (MIRU-VNTR) since 2009. NTSS collects clinical, demographic, and risk factor data for all reported tuberculosis cases in the United States. NTGS and NTSS data are linkable through unique case numbers.

We used NTGS and NTSS data for all genotyped cases reported during January 2009–December 2014 to attribute cases to recent transmission using a plausible source-case method [4]. Briefly, the method is based on the identification of a plausible source case, defined as a case with the same tuberculosis genotype, an infectious form of tuberculosis (e.g., pulmonary disease), occurring within 10 miles, and diagnosed within the 2 years before or the 3 months after the diagnosis of the secondary case. Thus, we searched for plausible source cases for all genotyped cases occurring during January 2011–September 2014. Cases from 49 states and the District of Columbia were included in analysis; cases from Oklahoma were excluded because they lacked sufficient geographic data to apply the plausible source-case method.

Starting from the source-secondary case pairs identified by the plausible source case method, we grouped cases into transmission clusters using social network analysis (NodeXL). A plausible source case and all secondary cases attributed to it were considered to be part of the same transmission cluster; a secondary case and all of its plausible source cases were also considered to be part of the same transmission cluster. Clusters were not restricted to jurisdictional boundaries.

We expected that situations in which transmission was limited, whether by tuberculosis control efforts or other factors, would differ from situations in which uncontrolled tuberculosis transmission occurred. To analyze these two situations separately, we used the sizes of the transmission clusters to classify cases as attributable to either limited recent transmission or extensive recent transmission. We defined the largest 10% of clusters as attributable to extensive recent transmission.

### Statistical Analysis

We determined the proportions of genotyped cases attributable to recent transmission by state and county. To assess geographic heterogeneity within states, we compared the proportion of genotyped cases attributed to recent transmission in a county to the proportion in the state where the county is located. To assess the association between tuberculosis incidence and recent transmission, we performed a simple linear regression to predict the proportion of genotyped cases attributed to recent transmission in a state based on state tuberculosis incidence, with observations weighted by the number of genotyped cases in each state.

We evaluated patient characteristics associated with recent transmission using logistic regression. For both bivariate and multivariable analyses, we separately analyzed factors associated with limited recent transmission and extensive recent transmission, using cases not attributed to recent transmission as the reference group. We performed sensitivity analysis using different transmission cluster size thresholds to define extensive versus limited recent transmission. We deferred to NTSS definitions of patient characteristics, which include U.S. Census definitions of self-reported race and ethnicity, and the classification of persons born in U.S. territories and affiliated islands as U.S.-born. Statistical analyses were performed using SAS version 9.3 (Cary, NC).

### Ethics Statement and Public Availability of Data

All data were collected and analyzed as part of routine public health surveillance. This project was therefore determined not to be human subjects research by the U.S. Centers for Disease Control and Prevention and did not require approval by an institutional review board.

The data contain information abstracted from the national tuberculosis case report form called the Report of Verified Case of Tuberculosis (RVCT) (OMB No. 0920–0026). These data have been reported voluntarily to CDC by state and local health departments, and are protected under the Assurance of Confidentiality (Sections 306 and 308(d) of the Public Health Service Act, 42 U.S.C. 242k and 242m(d)), which prevents disclosure of any information that could be used to directly or indirectly identify patients. For more information, see the CDC/ATSDR Policy on Releasing and Sharing Data (at http://www.cdc.gov/maso/Policy/ReleasingData.pdf). A limited dataset is available at http://wonder.cdc.gov/tb.html. Researchers seeking additional data may apply to analyze National TB Surveillance System data at CDC headquarters by contacting Dr. Thomas Navin (trn1@cdc.gov).

The findings and conclusions in this manuscript are those of the authors and do not necessarily represent the official position of the Centers for Disease Control and Prevention or the U.S. Department of Health and Human Services.

## Results

**Fig 1:**
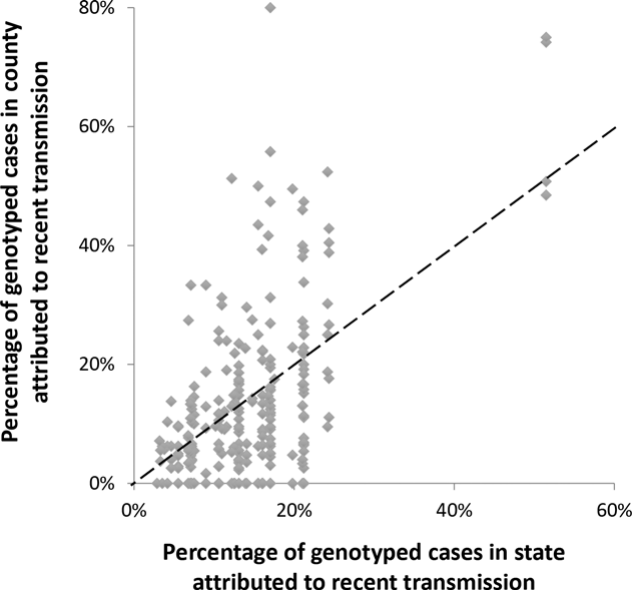
Proportion of genotyped cases attributed to recent transmission in each county compared to proportion of genotyped cases attributed to recent transmission in state where county is located—January 2011–September 2014. Each point represents one county. Only counties with ≥15 genotyped cases during the analysis period (i.e., an average of one case per quarter) are shown. Points would be expected to cluster around the dashed black line if every county within a given state had the same proportion of cases attributable to recent transmission as the entire state.

During January 2011–September 2014, 26,586 genotyped cases, representing 95% of all culture-positive cases, were reported to NTGS. Of these, 3,827 (14%) were attributed to recent transmission. This proportion was similar in all four years (data not shown). Among the 49 states included and the District of Columbia, the median proportion of cases attributed to recent transmission was 10%, with a range from 0% to 51%. However, within each state, the proportion of cases attributed to recent transmission varied widely by county (Fig 1). At a state level, the proportion of cases attributed to recent transmission increased by 1.3 percentage points for each 1 case/100,000 population increase in incidence rate (p = 0.018). However, only 9% of the variance in the proportion of cases attributed to recent transmission was explained by variation in incidence (adjusted R^2^ = 0.09). Five of the eight states with very low tuberculosis incidences (<1 case per 100,000 persons) in 2014 (5) had counties in which ≥20% of genotyped cases were attributed to recent transmission (data not shown).

**Fig 2:**
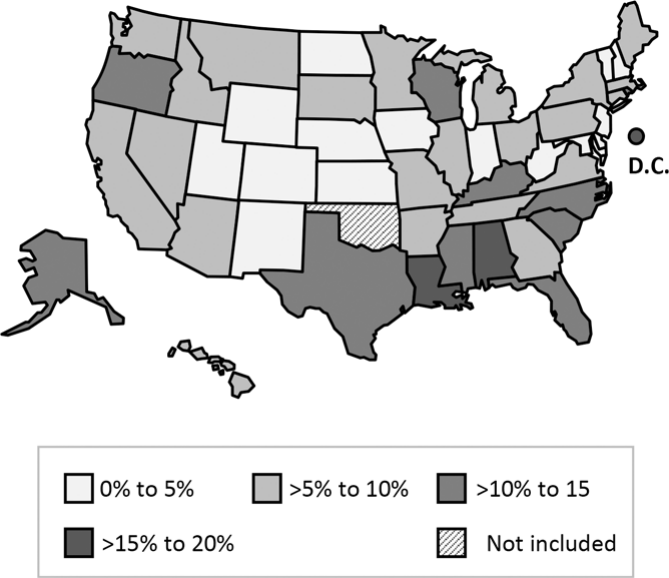
Map of states by proportion of cases attributed to limited recent transmission—January 2011–September 2014.

**Fig 3:**
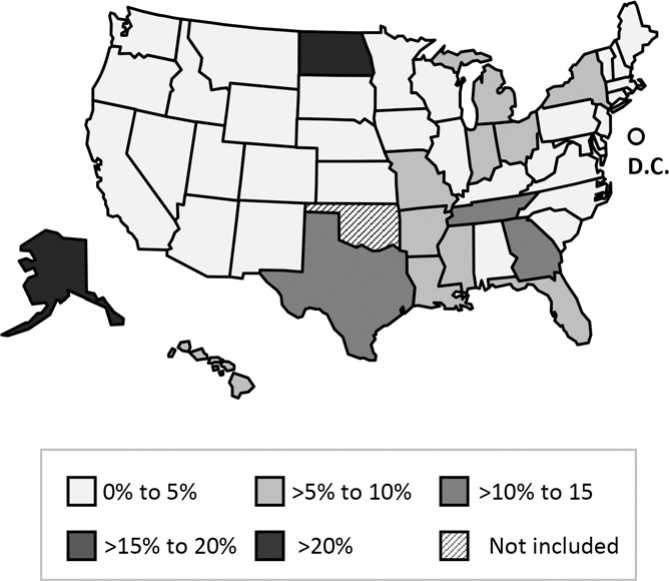
Map of states by proportion of cases attributed to extensive recent transmission—January 2011–September 2014.

In our analysis of transmission cluster sizes, 65% of clusters were observed to comprise two cases (i.e., one secondary case and its plausible source case). The largest decile of clusters comprised six or more cases. Therefore, we classified cases in transmission clusters of size ≤5 as attributable to limited recent transmission, and cases in transmission clusters of size ≥6 as attributable to extensive recent transmission. Of all cases attributed to recent transmission, 2,321 (61%) were classified as resulting from limited recent transmission, while 1,506 (39%) were classified as resulting from extensive recent transmission. Among states, the median proportion of cases attributed to limited recent transmission was 5% (range 0–20%) (Fig 2) and the median proportion of cases attributed to extensive recent transmission was 1% (range 0–40%) (Fig 3).

Table 1 shows patient characteristics of cases attributed and not attributed to recent transmission, with additional stratification of the latter group into cases resulting from limited versus extensive recent transmission. The characteristic showing the greatest magnitude of positive association with limited recent transmission was age ≤4 years (prevalence ratio [PR] = 5.1, 95% confidence interval [CI] 4.4–6.0) (Table 2). The characteristics showing the greatest magnitude of positive association with extensive recent transmission were American Indian/Alaska Native race (PR = 6.4, 95% CI 5.1–8.0) and homelessness (PR = 5.7, 95% CI 5.1–6.3).

**Table 1:** Frequency of patient characteristics among cases not attributed to recent transmission (N = 22,759) and attributed to recent transmission (N = 3,827).

Characteristic	Value	Not recent transmission		All recent transmission		Limited recent transmission		Extensive recent transmission	
		(N = 22,759)		(N = 3,827)		(N = 2,321)		(N = 1,506)	
		n	(%)	n	(%)	n	(%)	n	(%)
Sex	Female	8,758	(87.6)	1,244	(12.4)	812	(8.1)	432	(4.3)
	Male	13,998	(84.4)	2,582	(15.6)	1,508	(9.1)	1,074	(6.5)
Age (years)	≤4	129	(47.1)	145	(52.9)	112	(40.9)	33	(12.0)
	5–14	169	(73.8)	60	(26.2)	40	(17.5)	20	(8.7)
	15–24	2,246	(80.6)	542	(19.4)	364	(13.1)	178	(6.4)
	25–44	7,265	(86.1)	1,169	(13.9)	728	(8.6)	441	(5.2)
	45–64	6,919	(82.6)	1,454	(17.4)	785	(9.4)	669	(8.0)
	≥65	6,029	(93.0)	457	(7.0)	292	(4.5)	165	(2.5)
Origin	Foreign-born	16,065	(92.5)	1,298	(7.5)	937	(5.4)	361	(2.1)
	U.S.-born	6,677	(72.6)	2,522	(27.4)	1,382	(15.0)	1,140	(12.4)
Race and ethnicity*	Hispanic	6,547	(86.4)	1,035	(13.6)	729	(9.6)	306	(4.0)
	American Indian / Alaska Native	216	(60.2)	143	(39.8)	50	(13.9)	93	(25.9)
	Asian	7,736	(92.9)	591	(7.1)	387	(4.7)	204	(2.4)
	Black	4,279	(74.9)	1,438	(25.1)	746	(13.1)	692	(12.1)
	Native Hawaiian / Pacific Islander	127	(69.8)	55	(30.2)	27	(14.8)	28	(15.4)
	White	3,443	(86.7)	529	(13.3)	356	(9.0)	173	(4.3)
	Multiple races	375	(92.8)	29	(7.2)	23	(5.7)	6	(1.5)
Incarcerated at diagnosis	Yes	862	(82.7)	180	(17.3)	95	(9.1)	85	(8.2)
	No	21,835	(85.7)	3,633	(14.3)	2,218	(8.7)	1,415	(5.6)
	Unknown	62	(81.6)	14	(18.4)	8	(10.5)	6	(7.9)
Homeless	Yes	981	(62.5)	588	(37.5)	205	(13.1)	383	(24.4)
	No	21,596	(87.0)	3,216	(13.0)	2,099	(8.5)	1,117	(4.5)
	Unknown	182	(88.8)	23	(11.2)	17	(8.3)	6	(2.9)
Excess alcohol use	Yes	2,455	(72.9)	913	(27.1)	444	(13.2)	469	(13.9)
	No	19,961	(87.5)	2,859	(12.5)	1,841	(8.1)	1,018	(4.5)
	Unknown	343	(86.2)	55	(13.8)	36	(9.1)	19	(4.8)
Illicit substance use	Yes	1,450	(66.7)	724	(33.3)	352	(16.2)	372	(17.1)
	No	20,944	(87.3)	3,049	(12.7)	1,939	(8.1)	1,110	(4.6)
	Unknown	365	(87.1)	54	(12.9)	30	(7.2)	24	(5.7)
HIV status	Positive	1,274	(77.6)	367	(22.4)	192	(11.7)	175	(10.7)
	Negative	18,416	(85.6)	3,088	(14.4)	1,884	(8.8)	1,204	(5.6)
	Unknown	3,069	(89.2)	372	(10.8)	245	(7.1)	127	(3.7)

Cases attributed to recent transmission are further stratified as attributable to limited recent transmission (N = 2,321), and to extensive recent transmission (N = 1,506). Row percentages are presented.

*Persons of Hispanic ethnicity might be of any race; non-Hispanic persons are categorized as Asian, black, white, American Indian/Alaska Native, Native Hawaiian or other Pacific Islander, or of multiple races.

**Table 2:** Bivariate associations between patient characteristics and recent transmission.

Characteristic	Value	Limited recent transmission		Extensive recent transmission	
		Crude PR	Wald 95% CI	Crude PR	Wald 95% CI
Sex	Female	Reference		Reference	
	Male	**1.1**	**1.1–1.2**	**1.5**	**1.4–1.7**
Age (years)	≤4	**5.1**	**4.4–6.0**	**3.6**	**2.6–4.9**
	5–14	**2.1**	**1.6–2.8**	**1.9**	**1.2–2.8**
	15–24	**1.5**	**1.4–1.7**	**1.3**	**1.1–1.5**
	25–44	Reference		Reference	
	45–64	**1.1**	**1.0–1.2**	**1.5**	**1.4–1.7**
	≥65	**0.5**	**0.4–0.6**	**0.5**	**0.4–0.6**
Origin	Foreign-born	**0.3**	**0.3–0.4**	**0.2**	**0.1–0.2**
	U.S.-born	Reference		Reference	
Race and ethnicity*	Hispanic	1.1	1.0–1.2	0.9	0.8–1.1
	American Indian / Alaska Native	**2.0**	**1.5–2.6**	**6.4**	**5.1–8.0**
	Asian	**0.5**	**0.4–0.6**	**0.5**	**0.4–0.7**
	Black	**1.6**	**1.4–1.8**	**2.9**	**2.5–3.4**
	Native Hawaiian / Pacific Islander	**1.9**	**1.3–2.7**	**3.8**	**2.6–5.5**
	White	Reference		Reference	
	Multiple races	**0.6**	**0.4–0.9**	**0.3**	**0.2–0.7**
Incarcerated at diagnosis	Yes	1.1	0.9–1.3	**1.5**	**1.2–1.8**
	No	Reference		Reference	
	Unknown	1.4	0.7–2.6	1.4	0.6–3.1
Homeless	Yes	**1.9**	**1.7–2.2**	**5.7**	**5.1–6.3**
	No	Reference		Reference	
	Unknown	0.9	0.6–1.5	0.6	0.2–1.3
Excess alcohol use	Yes	**1.8**	**1.6–2.0**	**3.3**	**3.0–3.7**
	No	Reference		Reference	
	Unknown	1.0	0.8–1.5	1.0	0.6–1.5
Illicit substance use	Yes	**2.3**	**2.1–2.6**	**4.1**	**3.6–4.5**
	No	Reference		Reference	
	Unknown	0.8	0.6–1.2	1.1	0.7–1.7
HIV status	Positive	**1.4**	**1.2–1.6**	**2.0**	**1.7–2.3**
	Negative	Reference		Reference	
	Unknown	**0.8**	**0.7–0.9**	**0.6**	**0.5–0.8**

Prevalence ratios (PR) and 95% confidence intervals (CI) are shown for cases attributed to limited or extensive recent transmission compared to cases not attributed to recent transmission.

*Persons of Hispanic ethnicity might be of any race; non-Hispanic persons are categorized as Asian, black, white, American Indian/Alaska Native, Native Hawaiian or other Pacific Islander, or of multiple races.

Bold text indicates statistically significant result.

Foreign birth had the greatest magnitude of negative association with both limited (PR = 0.3, 95% CI 0.3–0.4) and extensive (PR = 0.2, 95% CI 0.1–0.2) recent transmission (Table 2). When cases in foreign-born patients were stratified by years since patients’ arrival in the United States, similar associations were observed with limited recent transmission regardless of time since arrival (data not shown). However, cases attributable to extensive recent transmission were more likely to have occurred in persons who had arrived in the United States >10 years before tuberculosis diagnosis (PR compared to U.S.-born patients = 0.2, 95% CI 0.2–0.2) than in persons who had arrived 1–5 years before diagnosis (PR compared to U.S.-born patients = 0.1, 95% CI 0.1–0.2).

The characteristic showing the greatest magnitude of independent positive association with limited recent transmission was age ≤4 years (adjusted prevalence ratio [aPR] = 2.8, 95% CI 2.4–3.3) (Table 3). The characteristics showing the greatest magnitude of independent positive association with extensive recent transmission were American Indian/Alaska Native race (aPR = 3.6, 95% CI 2.9–4.4), Native Hawaiian/Pacific Islander race (aPR = 3.2, 95% CI 2.3–4.5), black race (aPR = 3.0, 95% CI 2.6–3.5), Asian race (aPR = 2.4, 95% CI 1.9–3.0), and homelessness (aPR = 2.3, 95% 2.0–2.5). Foreign birth was negatively associated with both limited (aPR = 0.4, 95% CI 0.3–0.4) and extensive (aPR = 0.2, 95% CI 0.2–0.2) recent transmission.

**Table 3:** Multivariable models for associations between patient characteristics and limited or extensive recent transmission.

Characteristic	Value	Limited recent transmission		Extensive recent transmission	
		aPR	Wald 95% CI	aPR	Wald 95% CI
Sex	Female	Reference		Reference	
	Male	1.1	1.0–1.2	**1.1**	**1.0–1.3**
Age (years)	≤4	**2.8**	**2.4–3.3**	**1.9**	**1.4–2.6**
	5–14	**1.5**	**1.1–2.0**	1.3	0.8–1.9
	15–24	**1.4**	**1.3–1.6**	**1.2**	**1.1–1.5**
	25–44	Reference		Reference	
	45–64	0.9	0.8–1.0	1.0	0.9–1.1
	≥65	**0.5**	**0.4–0.6**	**0.5**	**0.4–0.6**
Origin	Foreign-born	**0.4**	**0.3–0.4**	**0.2**	**0.2–0.2**
	U.S.-born	Reference		Reference	
Race and ethnicity*	Hispanic	**1.5**	**1.3–1.7**	**2.0**	**1.7–2.4**
	American Indian /Alaska Native	**1.5**	**1.2–2.0**	**3.6**	**2.9–4.4**
	Asian	1.1	0.9–1.3	**2.4**	**1.9–3.0**
	Black	**1.6**	**1.4–1.8**	**3.0**	**2.6–3.5**
	Native Hawaiian / Pacific Islander	**1.5**	**1.1–2.2**	**3.2**	**2.3–4.5**
	White	Reference		Reference	
	Multiple races	1.0	0.7–1.5	0.9	0.4–1.9
Incarcerated at diagnosis	Yes	**0.8**	**0.6–0.9**	0.9	0.8–1.1
	No	Reference		Reference	
	Unknown	1.4	0.7–2.6	1.2	0.7–2.3
Homeless	Yes	**1.2**	**1.0–1.4**	**2.3**	**2.0–2.5**
	No	Reference		Reference	
	Unknown	1.0	0.6–1.7	0.7	0.3–1.6
Excess alcohol use	Yes	**1.2**	**1.1–1.3**	**1.2**	**1.1–1.4**
	No	Reference		Reference	
	Unknown	1.5	1.0–2.3	1.0	0.6–1.8
Illicit substance use	Yes	**1.2**	**1.1–1.4**	**1.2**	**1.1–1.4**
	No	Reference		Reference	
	Unknown	0.6	0.4–1.0	0.8	0.5–1.3
HIV status	Positive	1.1	0.9–1.2	1.1	0.9–1.2
	Negative	Reference		Reference	
	Unknown	0.9	0.8–1.1	0.9	0.7–1.1

Adjusted prevalence ratios (aPR) and 95% confidence intervals (CI) are shown for cases attributed to limited or extensive recent transmission compared to cases not attributed to recent transmission.

*Persons of Hispanic ethnicity might be of any race; non-Hispanic persons are categorized as Asian, black, white, American Indian/Alaska Native, Native Hawaiian or other Pacific Islander, or of multiple races.

Bold text indicates statistically significant result.

Changing the cluster size threshold for defining extensive recent transmission produced similar results in multivariable analysis for sex, foreign versus U.S. birth, HIV status, and most social risk factors. However, as the threshold was increased from three to six cases per cluster, cases resulting from extensive transmission were increasingly more likely to occur among persons experiencing homelessness and certain racial/ethnic minorities. In addition, as the threshold was increased, the magnitude of association between limited recent transmission and age ≤4 years decreased. Full results of this sensitivity analysis are provided as supporting information (S1 Table).

## Discussion

Based on a field-validated method for estimating cases resulting from recent transmission [4], we found that during January 2011–September 2014, 14% of genotyped tuberculosis cases in the United States were attributable to recent transmission. Of these, 39% were categorized as resulting from extensive recent transmission based on their being part of a transmission cluster of at least six cases. We observed substantial geographic heterogeneity in the proportion of cases attributed to recent transmission. Compared to cases not attributed to recent transmission, cases resulting from extensive recent transmission were more likely to occur in persons who belonged to racial minorities and persons experiencing homelessness. Cases resulting from limited recent transmission were more likely to occur in young children. However, cases resulting from both extensive and limited recent transmission were more likely to occur among U.S.-born persons than cases not attributed to recent transmission.

The proportion of cases attributable to recent transmission (14%) that we observed using the plausible source-case method is substantially lower than proportions of all genotype-clustered cases reported in the United States during 2012–2014 (22%) [5] or observed in previous studies of U.S. populations [2, 6, 7]. One explanation for this difference is that we defined recent transmission using a method validated by field epidemiology that takes into account genotype, geographic distance, time of diagnosis, and infectiousness of potential source cases, which has a calculated accuracy of 94% [4]. In contrast, most methods of defining genotype clusters do not require identification of plausible source cases, and their accuracy is unknown. Nevertheless, several risk factors for recent transmission identified in our analysis were similar to those identified in studies of genotypic clustering, including homelessness, racial/ethnic minority status, and U.S. birth [2, 3].

Our results illustrate the geographic heterogeneity of tuberculosis epidemiology in the United States. Not only did the proportion of cases attributed to recent transmission vary across states, but within a single state, some counties had substantial proportions of cases attributed to recent transmission while others had none. In addition, some states with very low tuberculosis incidence had counties with high levels of recent transmission. And at the state level, tuberculosis incidence alone was a weak predictor of the proportion of cases attributed to recent transmission. The fact that tuberculosis transmission is an issue in low-incidence states may be partially attributable to the limited capacity of health departments in these states, which receive very little federal funding for tuberculosis control, to carry out core activities such as contact investigations and targeted testing. Thus, our results suggest the importance of maintaining the capacity to respond to transmission in lower-incidence as well as high-incidence settings.

We observed that even though foreign-born persons accounted for over 60% of all tuberculosis cases in the United States during the study period [5], only 8% of cases attributed to recent transmission occurred among foreign-born persons. Furthermore, although the risk of having tuberculosis attributed to extensive recent transmission was higher for foreign-born persons who had been in the United States for longer, even those who had been in the United States for over a decade had substantially lower risk than U.S.-born persons. These findings, along with the observation that immigrants’ risk of tuberculosis diagnosis in the United States is associated with tuberculosis incidence in their countries of origin [8], suggests that most cases among foreign-born persons result from infection acquired prior to immigration. In addition, a higher index of suspicion for diagnosing tuberculosis in recent immigrants may help to ensure that those who develop tuberculosis are diagnosed and treated early, preventing additional transmission in immigrant communities. Thus, while the majority of tuberculosis cases in the United States occur among foreign-born persons [5], interventions to prevent tuberculosis transmission among U.S.-born populations should be strengthened to reduce ongoing transmission within the United States.

Our results suggest that cases resulting from limited recent transmission have different characteristics than cases resulting from extensive recent transmission. Patient characteristics associated with extensive recent transmission in our analysis have previously been identified among patients involved in tuberculosis outbreaks in the United States. For example, during 2002–2008, 91% of all patients in outbreaks investigated by CDC were U.S.-born and 67% were of non-Hispanic black race/ethnicity [9]. In comparison, during this time period, only 44% of all tuberculosis patients in the United States were U.S.-born and 28% were of non-Hispanic black race/ethnicity [5]. Outbreaks among American Indian and Alaska Native populations have also occurred [10, 11], and the persistent high rates of tuberculosis among these populations are at least partially a historic legacy of poverty and neglected and under-resourced health systems [12, 13]. Thus, our results serve as a warning that tuberculosis transmission and outbreaks may not subside unless health disparities in underserved groups in the United States are addressed.

Numerous outbreaks have also been reported among persons experiencing homelessness [14–17], and genotype clusters in which any of the initial three patients reported either homelessness or other social risk factors are at increased risk for growing into outbreaks in the United States [18]. People experiencing homelessness often have other risk factors for tuberculosis exposure and transmission, such as recent incarceration, substance abuse, and the use of homeless shelters [19]. Given this combination of factors, preventing tuberculosis transmission among homeless populations will require collaborations among public health departments, homeless shelters, and service providers. Coordinated screening for tuberculosis in homeless shelter, linkage to care for those diagnosed with tuberculosis disease or infection, and contact investigations around identified cases are all necessary to interrupt transmission.

While patient characteristics associated with extensive recent transmission echoed the characteristics observed among outbreak-related patients, limited recent transmission had the greatest magnitude of positive association with age under 5 years. We observed only slight associations between limited recent transmission and social risk factors including homelessness, as well as a negative association between limited recent transmission and incarceration. Together, these results are consistent with the hypothesis that cases attributed to limited recent transmission largely reflect household transmission, which can best be addressed by routine contact investigations.

The social risk factors of illicit substance use and excess alcohol use showed the same modest magnitude of independent association with both limited and extensive recent transmission. One contributing factor to these results could be immune system dysfunction or suppression associated with alcohol or drug use [20, 21], which could increase individuals’ risk of disease progression upon infection. Other contributing factors might be environmental, as transmission has been documented among social networks defined by substance use, potentially facilitated by the enclosed environments where these activities took place [22, 23].

The finding that HIV infection was not independently associated with cases resulting from recent transmission was counterintuitive, given that HIV is known to increase a person’s risk of developing tuberculosis once infected [24], and that the resurgence of tuberculosis in the United States during the late 1980’s and early 1990’s was partially attributed to the HIV epidemic [25]. It is possible that by 2009, tuberculosis rates in the United States might have declined to the extent that people with HIV were not at high enough risk of tuberculosis exposure to result in a significant independent association with recent tuberculosis transmission.

Our analysis was subject to limitations. First, cases resulting from recent transmission cannot definitively be differentiated from those caused by reactivation. While the method used to attribute cases to recent transmission has been validated using epidemiologic data and optimized for sensitivity and specificity [4], misclassification errors may have occurred. Second, because genotyping can only be performed for cases with a cultured isolate of *Mycobacterium tuberculosis* complex, our results may not be generalizable to all tuberculosis patients, as only 77% of tuberculosis cases in 2014 were confirmed by culture [5]. Third, because belonging to a transmission cluster was the outcome variable in our analysis of patient-level predictors of recent transmission, we were unable to statistically account for the possibility that patients within a transmission cluster might be more similar to each other than patients not in the same cluster; thus, our confidence intervals may be artificially narrow due to correlated data. Fourth, because we lacked data on patients’ socioeconomic status, we were unable to determine the extent to which associations with race and ethnicity were driven by socioeconomic factors. And fifth, we were unable to independently assess the effect of mycobacterial strain or lineage in this analysis because in the United States, many strains are closely associated with population groups, and our routine surveillance data does not allow us to control for all the characteristics that define these populations.

One final limitation is that our distinction between limited and extensive recent transmission was not based on any standard definition, but rather a hypothesis that populations in which transmission is effectively limited by tuberculosis control efforts would differ from populations in which uncontrolled tuberculosis transmission is more common. We chose a relatively large transmission cluster size as a threshold for extensive recent transmission to identify more specifically characteristics of this latter population. Indeed, the results of our sensitivity analysis suggested that increasing the threshold defining extensive recent transmission accentuated the distinction between cases resulting from extensive recent transmission compared to those resulting from limited recent transmission.

In conclusion, applying a field-validated method to U.S. tuberculosis surveillance data indicated that the proportion of tuberculosis cases resulting from recent transmission may be lower than previous estimates have suggested. However, the contribution of transmission to overall tuberculosis case burden varies geographically, and transmission can be a major public health issue even in states with low incidences of tuberculosis. Finally, the patient characteristics associated with extensive recent transmission, such as homelessness, birth in the United States, and racial/ethnic minority groups, suggest higher-risk populations in which to focus interventions to identify, prevent, and halt tuberculosis outbreaks.

## Supporting Information

S1 Table Results of sensitivity analysis showing impact of changing cluster size threshold. Adjusted prevalence ratios (aPR) and 95% confidence intervals (CI) are shown for cases attributed to limited or extensive recent transmission compared to cases not attributed to recent transmission.
(XLS)
